# A Comparison of Three Different Bioinformatics Analyses of the 16S–23S rRNA Encoding Region for Bacterial Identification

**DOI:** 10.3389/fmicb.2019.00620

**Published:** 2019-04-16

**Authors:** Nilay Peker, Sharron Garcia-Croes, Brigitte Dijkhuizen, Henry H. Wiersma, Evert van Zanten, Guido Wisselink, Alex W. Friedrich, Mirjam Kooistra-Smid, Bhanu Sinha, John W. A. Rossen, Natacha Couto

**Affiliations:** ^1^Department of Medical Microbiology and Infection Prevention, University Medical Center Groningen, University of Groningen, Groningen, Netherlands; ^2^Department of Medical Microbiology, Certe, Groningen, Netherlands

**Keywords:** clinical microbiology, diagnostics, next-generation sequencing, metagenomics, OTU clustering, mapping, *de novo* assembly

## Abstract

Rapid and reliable identification of bacterial pathogens directly from patient samples is required for optimizing antimicrobial therapy. Although Sanger sequencing of the 16S ribosomal RNA (rRNA) gene is used as a molecular method, species identification and discrimination is not always achievable for bacteria as their 16S rRNA genes have sometimes high sequence homology. Recently, next generation sequencing (NGS) of the 16S–23S rRNA encoding region has been proposed for reliable identification of pathogens directly from patient samples. However, data analysis is laborious and time-consuming and a database for the complete 16S–23S rRNA encoding region is not available. Therefore, a better, faster, and stronger approach is needed for NGS data analysis of the 16S–23S rRNA encoding region. We compared speed and diagnostic accuracy of different data analysis approaches: *de novo* assembly followed by Basic Local Alignment Search Tool (BLAST), operational taxonomic unit (OTU) clustering, or mapping using an in-house developed 16S–23S rRNA encoding region database for the identification of bacterial species. *De novo* assembly followed by BLAST using the in-house database was superior to the other methods, resulting in the shortest turnaround time (2 h and 5 min), approximately 2 h less than OTU clustering and 4.5 h less than mapping, and a sensitivity of 80%. Mapping was the slowest and most laborious data analysis approach with a sensitivity of 60%, whereas OTU clustering was the least laborious approach with 70% sensitivity. Although the in-house database requires more sequence entries to improve the sensitivity, the combination of *de novo* assembly and BLAST currently appears to be the optimal approach for data analysis.

## Introduction

Clinical microbiology strives to improve patient care by rapidly identifying and characterizing microbial pathogens in patient samples to establish a correct diagnosis and to ensure optimal treatment and infection prevention. The conventional culture has long been considered the gold standard for bacterial identification. However, it can take days to weeks to successfully culture bacteria, as some clinically relevant bacteria are slow-growing, difficult to grow, fastidious or sometimes even non-culturable (Didelot et al., 2012; Salipante et al., 2013). As a complementary approach to culture, sequencing of the 16S rRNA gene has emerged as an accurate and faster method widely used for bacterial identification (Petti, 2007; Srinivasan et al., 2015). The 16S rRNA gene, ∼1.5 kilobase (kb) in length, has proven to be a useful molecular target since it is present in all bacteria, either as a single copy or in multiple copies, and it is highly conserved over time within a species (Petti, 2007; Sabat et al., 2017). However, this method does not always allow to identify bacteria to the species level due to high sequence similarities between some species (Deurenberg et al., 2017). For example, species of the *Streptococcus mitis* group, including *Streptococcus pneumoniae*, are almost indistinguishable from each other on the basis of their 16S rRNA genes, having 99–100% sequence similarities (Petti, 2007; Lal et al., 2011). This method also fails to distinguish certain species, as described for *Escherichia coli* and *Shigella* spp. the 16S rRNA genes of which share >99% sequence identity (Devanga Ragupathi et al., 2017). Furthermore, Sanger sequencing, which is generally used for 16S rRNA gene sequencing, is challenging in complex, polymicrobial samples (Deurenberg et al., 2017). With the continuous advancements in sequencing technology over the past decade, next-generation sequencing (NGS) offers several advantages over Sanger sequencing, including a higher resolution and accuracy in identifying microbial pathogens (MacCannell, 2016; Motro and Moran-Gilad, 2017). Moreover, this technology allows culture-independent testing from complex polymicrobial samples to detect and identify several pathogens in parallel (Rossen et al., 2018). A diagnostic method based on NGS of PCR amplification products of the 16S–23S rRNA encoding region (∼4.5 kb) has been developed (Benítez-Páez and Sanz, 2017; Kerkhof et al., 2017), showing a higher resolution and a reduced time to results for bacterial identification compared to other identification methods (e.g., 16S rRNA gene Sanger sequencing) (Sabat et al., 2017). However, this method had some limitations, including the absence of an extensive 16S–23S rRNA encoding region database and the lack of complementary software allowing easy and reliable species identification (Sabat et al., 2017).

Operational taxonomic unit (OTU) clustering is a widely used tool to identify the bacterial composition of a sample based on the 16S rRNA gene sequencing. *De novo* assembly followed by BLASTN or mapping, on the other hand, are commonly used for the analysis of whole genome sequencing data. *De novo* assembly followed by BLASTN on the NCBI database has been described as the main tool used for the bacterial identification from clinical samples based on NGS of the 16S–23S rRNA encoding region (Sabat et al., 2017). In a later study, where Nanopore sequencing of the 16S–23S rRNA encoding region was performed, a mapping based approach was used to analyze the data (Cuscó et al., 2018). However, so far it is unclear which method is more accurate and/or faster. Therefore, we have performed the data analysis of 16S–23S rRNA encoding region using the tools for 16S rRNA gene sequencing and whole genome sequencing to find out the most appropriate tool in terms of diagnostic accuracy and speed. We first evaluated different sequencing protocols for NGS of the 16S–23S rRNA encoding region and subsequently tested the speed and diagnostic accuracy of three different 16S–23S rRNA encoding region NGS data analyses, *de novo* assembly followed by BLASTN, mapping and OTU clustering for the correct assignment of bacterial species directly from clinical samples. In order to accomplish this objective, we used culture and 16S rRNA gene Sanger sequencing as gold standard.

## Materials and Methods

### Samples

Twenty heart valve tissues and eight fluid clinical samples (five fluids from sonicated valve tissues, three pus and liquor) presented to the medical microbiology laboratory for routine diagnostics (culturing and 16S Sanger sequencing) were in parallel tested by the 16S–23S rRNA encoding region NGS approach. The samples used for the present analyses were collected during routine diagnostics. All procedures were carried out according to guidelines and regulations of University Medical Center Groningen (UMCG) concerning the use of patient materials for the validation of clinical methods, which follow the guidelines of the Federation of Dutch Medical Scientific Societies (FDMSS). Every patient entering the UMCG is informed that samples taken may be used for research and publication purposes, unless they indicate that they do not agree to it. This procedure has been approved by the Medical Ethical Committee of the UMCG. All samples were used after performing and completing conventional microbiological diagnostics and were coded to protect patient confidentiality.

In addition, a mock community sample, i.e., the ZymoBIOMICS^TM^ Microbial Community DNA Standard (Zymo Research, Irvine, CA, United States), was used for NGS of the 16S–23S rRNA encoding region. The mock community consisted of the following eight bacterial species: *Bacillus subtilis, Enterococcus faecalis, Escherichia coli, Lactobacillus fermentum, Listeria monocytogenes, Pseudomonas aeruginosa, Salmonella enterica, Staphylococcus aureus*.

### Culturing

Samples were cultured on 5% sheep blood agar (BA), chocolate agar (CHOC), Fastidious Broth (FB) and/or Brucella blood agar (BBA) plates (Mediaproducts BV, Groningen, Netherlands). The BA and CHOC agar plates were aerobically (with 5% CO_2_) at 35°C, and BBA and FB agar plates were anaerobically incubated at 35°C up to 9 days. When growth was recorded, the identification was done by matrix-assisted laser desorption ionization time-of-flight mass spectrometer (MALDI-TOF MS) (Bruker, Billerica, MA, United States).

### DNA Extraction From Clinical Samples

The DNA from tissues and fluid samples was extracted and purified using the DNeasy Blood and Tissue Kit (Qiagen, Hilden, Germany) for 16S rDNA sequencing and the PureLink^TM^ Genomic DNA Mini Kit (Thermo Fisher, Bleiswijk, Netherlands) for 16S–23S rRNA encoding region NGS, according to the manufacturers’ protocols.

### Sanger Sequencing of the 16S rRNA Gene

Extracted DNA from the clinical samples was amplified by PCR using 16S rRNA gene targeting primers 8F (5′-TGGAGAGTTTGATCCTGGCTCAG-3′) and 515R (5′-TACCGCGGCTGCTGCTGGCAC-3′) (Biolegio, Nijmegen, Netherlands). PCR was performed on the T100 Thermal Cycler (Bio-Rad) with the following conditions: initial incubation for 15 min at 95°C followed by 35 cycles of 15 s at 94°C, 15 s at 60°C, 30 s at 72°C with a final incubation for 10 min at 72°C. In the following, DNA sequencing was performed with an automated DNA sequencer (ABI 3130XL; Applied Biosystems Instrument, Carlsbad, CA, United States) using the BigDye Terminator v3.1 cycle sequencing kit. The sequencing data was analyzed using SeqMan Pro v10.0.1 (DNASTAR, Madison, WI, United States) by assembling the forward and reverse reads into a consensus sequence. Subsequently, the consensus sequences were aligned in the GenBank database using the web-based basic local alignment tool (BLAST).

### Next-Generation Sequencing of the 16S–23S rRNA Encoding Region

Extracted DNA from the clinical samples and of the mock community sample was quantified using Qubit^®^ 2.0 Fluorometer (Thermo Fisher, Bleiswijk, Netherlands) by following the manufacturer’s instructions. PCR amplification of the 16S–23S rRNA encoding region was performed using the forward primer 27F (5′-AGAGTTTGATCMTGGCTCAG-3′), targeting the 16S rRNA gene and the reverse primer 2490R (5′-GACATCGAGGTGCCAAAC-3′) (Applied Biosystems UK, Renfrewshire, United Kingdom) targeting the 23S rRNA gene, for extracted DNA samples as well as for the negative control, which consisted of only RNA- and DNA-free water, and for the positive control (containing DNA from *Delftia lacustris*, DSMZ 21246). The amplification was carried out as previously described (Sabat et al., 2017), with the following minor modifications: for the reaction mixture, 200 μM nucleotide mix dNTPs (Roche Diagnostics, Almere, Netherlands) was used and 35 cycles consisting of incubation at 98°C for 30 s, followed by incubation at 70°C for 30 s and at 72°C for 2 min. PCR products were analyzed using the Agilent D500 ScreenTape kit (Agilent Technologies Netherlands B.V., Amstelveen, Netherlands) according to manufacturer’s protocol using the 2200 TapeStation System (Agilent Technologies). Subsequently, PCR products were purified and quantified and NGS libraries were prepared using the Nextera XT DNA Library Preparation Kit (Illumina, San Diego, CA, United States). Next, each library was normalized, pooled and loaded onto the Illumina MiSeq platform for paired-end sequencing. For the evaluation of three different sequencing protocols, firstly 11 of the clinical samples were paired-end sequenced using either a 300-cycles MiSeq Reagent Kit V2 (300_v2) (Illumina), a 500-cycles MiSeq Reagent Kit V2 (500_v2) (Illumina) or a 600-cycles MiSeq Reagent Kit V3 (600_v3) (Illumina). After determining the optimal protocol, which was the 300_v2, the other clinical samples were sequenced using the 300_v2 sequencing kit (Illumina).

### 16S–23S rRNA Encoding Region Database

For the creation of the 16S–23S rRNA encoding region sequence database, sequences of a minimum of two strains per bacterial species were used. Sequences of each strain were obtained as a FASTA file from the NCBI genome website^1^ by using the chromosome coordinates on the chromosome NCBI Reference Sequence. If no chromosome NCBI Reference Sequence was available, the whole-genome sequences of the corresponding species were taken as a GenBank file and annotated on the Rapid Annotation Subsystem Technology (RAST) server^2^. After annotation, the 16S–23S rRNA encoding region coordinates were used to download the FASTA file from the NCBI genome website. All obtained 16S–23S rRNA encoding region sequences were concatenated into one multiple FASTA file using command line on Mac OS X, a Unix based operating system.

To expand the database, 16S–23S rRNA encoding region sequences presented in a recently published study (Benítez-Páez and Sanz, 2017) were downloaded and merged using a scripting language, Python (version 3.6.2) to generate our final taxonomy and mapping database. For this, duplicate sequences were filtered out from our FASTA file containing multiple 16S–23S rRNA encoding region sequences (*mff*) (*n* = 176 bacterial species) by comparing it to the FASTA file of 16S–23S rRNA encoding region sequences (ss) obtained from the previously published database (Benítez-Páez and Sanz, 2017) (*n* = 2339 bacterial species) and a multiple FASTA file with unique sequences was created. The published database was also curated in order to remove entries that did not contain a bacterial species/genus or contained taxonomic errors and remove entries that had the wrong length (e.g., length size >10,000 and <2,500). Then, two files: (i) the taxonomy dump file^3^ (accessed on 20-09-2017) and (ii) the lineage file^4^ (accessed on 20-09-2017) were used to get the lineage (taxonomy) for specific bacteria. Subsequently, the mapping database was created using the merged taxonomy text file and its corresponding sequencing multiple FASTA file. The database contained 23,439 sequences from 2389 species (295 sequences were not identified at the species level, and could represent new species) and 896 genera. The sequencing and taxonomy databases were annotated using the “Set up Amplicon-Based Reference Database” tool from the CLC Microbial Genomics Module (version 3.0). These were annotated to a similarity percentage of 99%, creating an OTU database compatible for OTU clustering.

### Data Analysis of the 16S–23S rRNA Encoding Region NGS

The FASTQ files containing the sequencing reads were analyzed using the CLC Genomics Workbench version 11.0 (Qiagen) (see Supplementary Table S1 describing the parameters used for data analysis). First, the paired-end reads were trimmed with 0.05 (corresponding to a Phred quality score Q ≥ 14) quality scores. We also performed adapter trimming using the Nextera XT adapters as references.

### *De novo* Assembly and BLAST

In the *de novo* assembly using CLC Genomics Workbench, contiguous sequences are generated through de Bruijn graph algorithms (Ekblom and Wolf, 2014). After *de novo* assembly, the generated contigs are assigned a taxonomic classification by alignment using the nucleotide Basic Local Alignment Search Tool (BLASTN) against the nucleotide collection database (NCBI database).

Following quality trimming, trimmed paired end reads were *de novo* assembled into contigs^5^. The contigs with a total read count of >1,000 reads were chosen and were aligned to (i) the nucleotide collection database on NCBI, using the web-based nucleotide BLAST and (ii) an in-house developed 16S–23S rRNA encoding region database for bacterial identification.

(i) The alignment on NCBI was manually performed by submitting contigs’ sequences via the website^6^. According to the similarity score defined previously (Sabat et al., 2017), bacteria could be assigned to a species or genus when the similarity score was ≥99% and between 90 and 99%, respectively. An identity score of <90% was interpreted as an unidentified microorganism. Furthermore, the contigs aligned on the NCBI database were filtered by setting the Expect value (E) to 0, and by excluding pathogens found in the negative control. If more than one contig was generated for the same species, the reads of all contigs belonging to the same species were added up and the relative abundance of that particular bacterial species in each sample was calculated by dividing the total read count of the corresponding contigs by the total number of reads in the sample.
(ii) For the alignment using the local nucleotide database, the in-house developed 16S–23S rRNA encoding region database was uploaded to CLC Genomics Workbench. Then the BLAST analysis was performed for the contigs with a total read count of >1,000 reads. Further analysis of the results was performed as stated above.

The data for the evaluation of the three different sequencing protocols was analyzed using only the *de novo* assembly approach followed by a BLAST using the NCBI database. To assess the optimum sequencing workflow for bacterial identification, the sequencing results of the three different paired-end sequencing chemistries were evaluated based on time to result, and bacterial species identified, considering their abundance and identity level (similarity score ≥99% or <99%).

### OTU Clustering

OTU clustering groups the reads into OTUs, which consist of representative sequences of pseudo-species, based on sequence similarities and assigns taxonomy to them (OTU, 2017).

The reads after trimming were analyzed using the CLC Microbial Genomics Module (version 3.0) for OTU clustering. Reference based OTU clustering was performed using an in-house developed 16S–23S rRNA encoding region database with a similarity score of 99%, closed reference OTU picking was selected and, the minimum occurrences was set to 10. Further analysis was done manually by aggregating all the generated OTU’s by their species name and by excluding the results found in the negative control from clinical samples. Then, the percentage of the total abundance was calculated by dividing the combined abundance of the species with the total reads in OTU’s.

### Mapping to an In-House Developed 16S–23S rRNA Encoding Region Database

The mapping consists of aligning reads to reference sequences based on a predefined length and similarity fraction (CLC, 2017).

Trimmed reads were analyzed using the Map Reads to Reference tool with the default settings (similarity fraction 0.8; length fraction 0.5; minimum consensus length 200 bp) by aligning the reads against the in-house developed 16S–23S rRNA encoding region database. After alignment, the results were filtered based on total number of reads >1,000. Further analysis was done manually by excluding the species found in the negative control and then by aggregating each reference sequence to its species’ name. Afterward, the proportion of mapped reads was calculated by summing up the total read count for each species and dividing it by the total of mapped reads of the given sample.

### Statistical Analysis

During the data analysis, the species found in the negative control were excluded from the clinical samples in all three approaches, and were considered as potential contaminating species. Then a cut-off value was determined for each method to define whether the bacterial species identified should be accounted as infectious causing pathogens (e.g., *Cutibacterium acnes*) or as contamination. For each method, the cut-off value was determined by calculating the mean (μ) and standard deviation (SD) of the proportion of reads belonging to each species in samples which were negative by both culturing and Sanger sequencing using the confidence interval method (Singh, 2006). The results of the three data analysis approaches were compared based on the number of the species identified, the relative abundance (number of reads for a specific species), and time to result.

Statistical analysis was performed for all three data analysis approaches using the software package SPSS version 23 (IBM Corporation, New York, NY, United States) in which sensitivity and specificity of each approaches was determined.

## Results

### Evaluation of Three Different Sequencing Protocols

**FIGURE 1:**
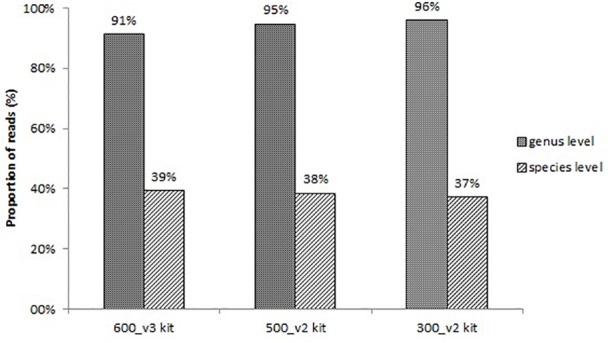
Proportion of reads corresponding to bacteria identified at the genus level and at the species level using three different sequencing protocols.

All sequencing protocols showed similar identification results at the species level. The proportion of reads corresponding to bacteria identified at the genus level and at the species level is shown in Figure 1. The remaining non-identified reads are belonged to other taxonomies (mainly human). The proportion of reads identified at the genus level was 91%, 95%, and 96%, and at the species level 39%, 38%, and 37% for the 600_v3 kit, 500_v2 kit, and 300_v2 kit, respectively (Figure 1). Despite the slightly lower proportion of reads identified at the species level, the 300_v2 kit identified the infection causing pathogen in all samples also identified using the other two sequencing kits. The main differences between sequencing kits were due to differences in the detection of contaminating species. On the other hand, compared to the 300_v2 kit (24 h) sequencing with the 600_v3 kit (56 h) took at least two times longer. Taking everything into account, sequencing using the 300_v2 kit was chosen as the sequencing protocol for the rest of the following samples.

### Data Analysis of All Clinical Samples

**FIGURE 2:**
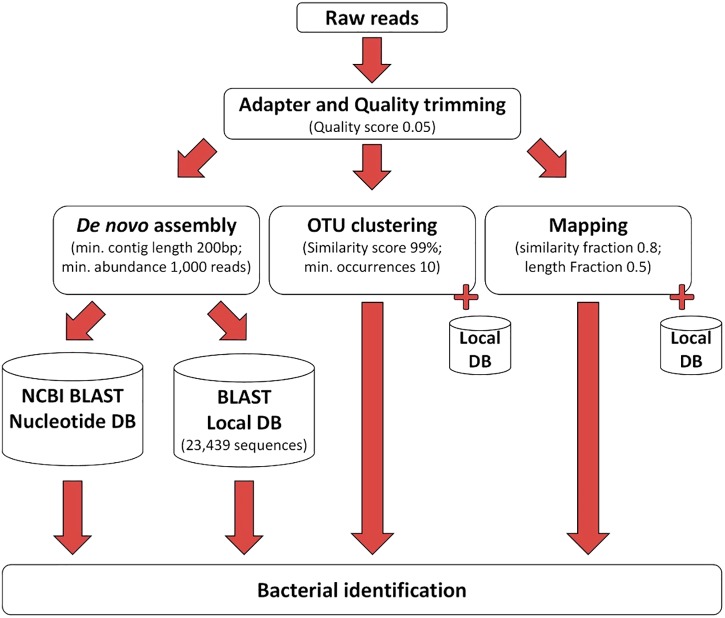
The workflow used for processing of the NGS data.

The processing of the sequencing reads obtained by the 16S–23S rRNA encoding region NGS method is illustrated in Figure 2. After removing the low-quality nucleotides by trimming, an average of 1,367,822 reads (99.9%) remained for analysis. Subsequent data analysis using the three different approaches shown in Figure 2 identified bacterial species in the negative control (Supplementary Table S2), and these species were considered to be contaminating species. If these species were found in clinical samples, they were excluded from further analysis unless they were above the cut-off level defined for each tool (Supplementary Tables S2–S4 and Tables 1, 2). From this point on, the samples identified with clinically relevant bacteria using either 16S Sanger sequencing or culturing were named conventional positive samples and those identified by using NGS of the 16S–23S rRNA encoding region were named NGS positive samples.

**Table 1:** Comparison of BLAST analysis results for NGS positive samples using the local database and the NCBI database.

		BLAST on local database		BLAST on NCBI database	
			
Sample	% of reads against contig	Species	% Identity	Species	% Identity
1^#^	0.5%	*Cutibacterium acnes*	100%	*Cutibacterium acnes*	100%
2^#^	0.4%	*Cutibacterium acnes*	100%	*Cutibacterium acnes*	100%
10^#^	0.4%	*Cutibacterium acnes*	100%	*Cutibacterium acnes*	98%
	0.7%	*Enterococcus faecium*	100%	*Enterococcus faecium*	100%
11^#^	0.4%	*Cutibacterium acnes*	100%	*Cutibacterium acnes*	99%
15^##^	0.4%	*Cutibacterium* sp.	93%	*Cutibacterium* sp.	93%
17^#^	0.7%	*Cutibacterium acnes*	100%	*Cutibacterium acnes*	99%
18^#^	95.8%	*Streptococcus* sp.	100%	*Streptococcus* sp.	99%
	0.32%	*Bordetella* sp.	96%	–	–
20^##^	74.3%	*Ureaplasma parvum*	100%	*Ureaplasma parvum*	100%
21^##^	61.1%	*Ureaplasma parvum*	100%	*Ureaplasma parvum*	99%
25^#^	88.9%	*Streptococcus dysgalactiae*	100%	*Streptococcus dysgalactiae*	100%
26^#^	80.2%	*Streptococcus sanguinis*	100%	*Streptococcus sanguinis*	99%
	2.0%	–	–	*Undibacterium oligocarboniphilum*	100%
27^##^	10.0%	*Actinotignum* sp.	97%	*Actinotignum sp.*	97%
	6.2%	*Aerococcus urinae*	100%	*Aerococcus urinae*	99%
	1.4%	*Actinotignum schaalii*	99%	*Actinotignum schaalii*	99%
	1.0%	*Cutibacterium acnes*	99%	*Cutibacterium* sp.	96%
Time^∗^	CLC analysis	∼1 h 20 min		∼1 h 20 min	
	Hands on	∼45 min		∼4 h	
	Total	∼2 h 5 min		∼5 h 20 min	


*^∗^Analysis time is for all 30 samples (including positive and negative control). ^#^Tissue sample; ^##^Fluid sample; *Cutibacterium acnes* had been formerly referred to as *Propionibacterium acnes*.*

**Table 2:** Bacterial species identified by NGS of 16S–23S rRNA encoding region using three different data analysis approaches and 16S rRNA gene Sanger sequencing and culturing.

		NGS of 16S–23S rRNA encoding region			Conventional methods	
			
		*De novo* assembly+BLAST (cut-off: 0.3%)	OTU clustering (cut-off: 0.2%)	Mapping (cut-off: 0.4%)	16S rRNA gene Sanger sequencing	Culturing
	**Sample number**	**Bacteria (relative abundance, %)**	**Bacteria (relative abundance, %)**	**Bacteria (relative abundance, %)**	**Bacteria**	**Bacteria**

	1^#^	*Cutibacterium acnes* (0.5%)	Negative	Negative	*Streptococcus* sp.	*Cutibacterium acnes* + <1 *saccharolyticus*^¥^
	3^#^	Negative	Negative	*Gemella* sp. (0.5%)	Negative	Negative
	6^##^	Negative	*Variovorax paradoxus* (0.3%)	Negative	Negative	Negative
	10^#^	*Enterococcus faecium* (0.7%)	*Enterococcus faecium* (1.9%)	Negative	*Tropheryma whipplei*	Negative
		*Cutibacterium acnes* (0.4%)				
	11^#^	*Cutibacterium acnes* (0.4%)	Negative	Negative	Negative	Negative
	15^##^	*Cutibacterium* sp. (0.4%)	Negative	Negative	Negative	Negative
	17^#^	*Cutibacterium acnes* (0.7%)	Negative	Negative	*Tropheryma whipplei*	Negative
	18^#^	*Streptococcus* sp. (95.8%)	*Streptococcus* sp. (52.4%)	*Streptococcus* sp. (48.1%)	*Streptococcus* sp.	Negative
		*Bordetella* sp. (0.32%)	*Streptococcus pneumoni ae* (9.2%)	*Streptococcus australis* (15.1%)		
			*Streptococcus australis* (8.4%)			
			*Streptococcus mitis* (7.7%)			
			*Streptococcus suis* (6.1%)			
			*Streptococcus parasanguinis* (5.4%)			
			*Streptococcus agalactiae* (3.0%)			
			*Streptococcus anginosus* (2.5%)			
			*Streptococcus ictaluri* (2.1%)			
			*Streptococcus cristatus* (1.6%)			
			*Streptococcus intermedius* (1.2%)			
	20^##^	*Ureaplasma parvum* (74.3%)	*Ureaplasma parvum* (79.8%)	*Ureaplasma parvum* (69.1%)	*Ureaplasma parvum*	Negative
			*Ureaplasma urealyticum* (16.6%)	*Ureaplasma urealyticum* (8.5%)		
	21^##^	*Ureaplasma parvum* (61.1%)	*Ureaplasma parvum* (78.9%)	*Ureaplasma parvum* (60.6%)	*Ureaplasma parvum*	Negative
			*Ureaplasma urealyticum* (15.9%)	*Ureaplasma urealyticum* (8.0%)		
	23^#^	Negative	Negative	Negative	Negative	*Cutibacterium acnes*<< 1
	24^#^	Negative	Negative	Negative	*Cutibacterium acnes*	Negative
	25^#^	*Streptococcus dysgalactiae* (88.9%)	*Streptococcus dysgalactiae* (85.3%)	*Streptococcus*	*Streptococcus*	Negative
				*dysgalactiae* (81.4%)	*dysgalactiae*	
			*Streptococcus pyogenes* (7.7%)	*Streptococcus pyogenes* (6.6%)		
			*Streptococcus agalactiae* (6.5%)	*Streptococcus canis* (1.5%)		
				*Streptococcus suis* (0.8%)		
				*Streptococcus ictaluri* (0.5%)		
	26^#^	*Streptococcus sanguinis* (80.2%)	*Streptococcus sanguinis* (75.9%)	*Streptococcus sanguinis* (65.8%)	*Streptococcus mitis*	Negative
			*Streptococcus anginosus* (8.6%)	*Streptococcus gordonii* (1.9%)		
			*Streptococcus cristatus* (3.8%)	*Streptococcus parasanguinis* (1.1%)		
			*Streptococcus parasanguinis* (3.6%)	*Streptococcus* sp. (1.0%)		
			*Streptococcus agalactiae* (2.6%)	*Streptococcus anginosus* (0.9%)		
			*Streptococcus pneumoniae* (1.6%)	*Streptococcus constellatus* (0.9%)		
			*Streptococcus constellatus* (0.8%)	*Streptococcus cristatus* (0.9%)		
			*Streptococcus gordonii* (0.4%)	*Streptococcus infantis* (0.5%)		
	27^##^	*Actinotignum schaalii* (1.4%)	*Actinotignum schaalii* (17.5%)	*Actinotignum schaalii* (14.2%)	*Actinotignum schaalii*	Negative
		*Actinotignum* sp. (10.0%)	*Aerococcus urinae* (13.9%)	*Aerococcus urinae* (7.4%)		
		*Aerococcus urinae* (6.2%)				
		*Cutibacterium acnes* (1.0%)				

Time^∗^	CLC analysis	∼1 h 20 min	∼3 h	∼2 h 30 min		
	Hands on	∼45 min	∼1 h	∼4 h		
	Total	∼2 h 5 min	∼4 h	∼6 h 30 min		


*^∗^Analysis time is for all 30 samples (including positive and negative control) using a i7-6700 CPU @ 3.40 GHz, 32 GB RAM, 64-bit operating system computer. ^¥^In later analysis, *Cutibacterium acnes* was identified. ^#^Tissue sample (heart valve); ^##^Fluid sample; *Cutibacterium acnes* had been formerly referred to as *Propionibacterium acnes*.*

#### Bacterial Identification on NCBI Database and Local 16S–23S rRNA Encoding Region Database Using BLAST Analysis

Bacterial identification results obtained by BLASTN analysis using both NCBI database and the local database are shown in Table 1. The same bacteria were identified at the genus and species level in all samples with two exceptions in samples 18 and 26. In sample 18, the contig with 454 bp (Supplementary Table S2) was identified as *Herbaspirillum* sp. using the NCBI database and was matching the partial sequence of the 16S rRNA gene with a similarity score of 96.47%, whereas the same contig was identified as *Bordetella* sp. (96% similarity score) using the local database. Likewise, in sample 26, the contig with 623 bp (Supplementary Table S2) was assigned as *Undibacterium oligocarboniphilum* in the NCBI database, could not be identified in the local database. The bacterial species in most of the samples were found with a slightly higher similarity score in the local database than in the NCBI database. We concluded that the local database was accurate enough to identify and distinguish clinically relevant species. Moreover, the time to complete the analysis of 30 samples was about 3 h more with BLAST on the NCBI database than the BLASTN on the local database (Table 1). Therefore, the other two approaches (OTU clustering and mapping) were performed using the local database.

#### Conventional Methods Versus 16S–23S rRNA Encoding Region NGS

Table 2 shows the bacterial identification results obtained by conventional methods (culture and 16S rRNA gene Sanger sequencing) and by 16S–23S rRNA encoding region NGS. The conventional methods identified bacterial species in 12 out of 28 samples. Among them, two samples (samples 2 and 33) were positive only by culturing and nine samples (samples 10, 17, 18, 20, 21, 24, 25, 26, and 27) were positive only by 16S rDNA Sanger sequencing. The 16S–23S rRNA encoding region NGS method identified the same bacteria in 42% (5/12) of the conventional positive samples, at the species level (samples 18, 20, 21, 25, and 27) and one sample at the genus level (sample 26) using the three data analysis approaches (Table 2). A *C. acnes* strain identified by conventional methods in sample 24 could not be detected by 16S–23S rRNA encoding region NGS using any of the bioinformatics tools. Also, *Tropheryma whipplei* could not be identified in samples 10 and 17 using the 16S–23S rRNA encoding region NGS method. By comparing the primers with the 16S–23S rRNA encoding region sequences of this species, we realized the primers did not align with the target region. Therefore, these two samples were excluded from further statistical analysis.

The *de novo* assembly and subsequent BLASTN analysis on the local database identified clinically relevant bacteria in 10 out of 26 samples with a sensitivity and specificity of 80% and 88%, respectively (Table 3) in comparison to conventional methods. The same bacteria were identified at species level in 5 (samples 18, 20, 21, 25, 27) out of 10 NGS positive samples and at genus level in one sample (sample 26) between Sanger sequencing and 16S–23S rRNA encoding region NGS. Apart from them, *de novo* assembly and following BLASTN analysis identified low abundant *C. acnes* in sample 1 as it was detected by culturing (Table 2). This approach identified *C. acnes* in sample 2, in which *Staphylococcus saccharolyticus* was detected by culturing. However, by doing further analysis, we found that in a subsequent sample taken from the same patient, *C. acnes* was identified. Furthermore, BLAST analysis of 16S–23S rRNA encoding region NGS identified additional bacterial species in samples 18 and 27.

**Table 3:** Sensitivity and specificity for all three data analysis approaches^∗^.

	Sensitivity in % (95% CI)	Specificity in % (95% CI)
*De novo* assembly + BLAST	80 (44.4–97.5)	88 (61.6–98.5)
OTU clustering	70 (34.7–93.3)	94 (69.8–99.8)
Mapping	60 (26.2–87.8)	94 (69.8–99.8)


*^∗^Statistical analysis performed using 26 clinical samples. CI, confidence interval.*

The OTU clustering approach detected bacterial species in eight out of 26 clinical samples (Table 2) with a sensitivity of 70% and a specificity of 94% compared to conventional methods (Table 3). In sample 6, OTU clustering identified a low abundant (0.3%) *Variovorax paradoxus*, a species that was not detected by the other two approaches and by conventional methods. Additionally, there were more, closely related bacterial species, identified in six of the NGS positive samples using the OTU clustering method.

A total of seven samples were positive using the mapping approach (Table 2). The sensitivity and specificity of the approach were 60% and 94%, respectively (Table 3). As mentioned above, bacteria identified in five of the samples (samples 18, 20, 21, 25, 27) coincided with the results of conventional methods at the species level and in one sample (sample 26), at the genus level. On the other hand, sample 3 was identified as *Gemella* sp. with an abundance of 0.5% using the mapping approach while it was negative using conventional methods.

#### Comparison of Three Approaches

The 16S–23S rRNA encoding region NGS data analysis results using the three different approaches are presented in Table 2 for NGS positive samples, and in Supplementary Tables S2–S4 for all samples. In six NGS positive samples, the same species was identified as the most abundant one with all three data analysis approaches. Different from BLASTN analysis, the OTU clustering and mapping approaches exhibited poor discrimination power in identifying closely related species in five of those NGS positive samples (samples 18, 20, 21, 25, 26) (Table 2). On the other hand, only *de novo* assembly and BLASTN analysis could identify low abundant *C. acnes* in samples 1, 2, 10, 11, 15, and 17 where, OTU clustering identified *C. acnes* only in sample 2. Furthermore, *de novo* assembly and BLASTN analysis showed higher positive rate (80% vs. 60–70%) in bacterial identification compared to the other two approaches (Table 3).

The time to complete the analysis for *de novo* assembly and BLAST using the local database was about 2 h for all 30 samples (including positive and negative control) while it took around 4 h including 1 h of hands-on-time for the OTU clustering and about 6 h and 30 min including 4 h of hands-on-time for mapping.

All the species present in the mock community sample were identified by both the OTU clustering and *de novo* assembly and BLAST approaches, whereas mapping did not identify two of the bacterial species (Supplementary Table S5). In addition, the OTU clustering and *de novo* assembly and BLAST approaches identified one more bacterial genus/species (*Fusobacterium* sp. and *Bacillus amyloliquefaciens*, respectively), which are not present in the mock community. Mapping identified several additional species that were not present in the mock community sample (Supplementary Table S5).

## Discussion

Until now, the main tool used for bacterial identification based on NGS of the 16S–23S rRNA encoding region was *de novo* assembly followed by BLASTN on NCBI database (Sabat et al., 2017), however, there was no evidence that it would be the most accurate and/or fastest method available. The *de novo* assembly and BLASTN is the only approach of the three that works at the contig level, and both the OTU clustering and mapping are performed at the read level. Using the NCBI database for these two last approaches would have resulted in odd results, since the NCBI database includes sequences that do not belong to the 16S–23S rRNA encoding region, but that due to the small read length, would have homology with our reads and would have resulted in the creation of bizarre OTUs and mapping results. The OTU clustering and mapping approaches are usually used when a database containing the sequences of interest are known, hence the need for the creation of a 16S-ITS-23S rDNA database. Therefore, in this study, we first assessed the use of an in-house developed and curated 16S–23S rRNA encoding region database for the NGS data analysis compared to the NCBI database. Secondly, we compared three different NGS data analysis approaches, *de novo* assembly and BLAST, OTU clustering, and mapping in terms of their capacity to accurately and efficiently identify bacterial species using the in-house developed 16S–23S rRNA encoding region database. The results show that the *de novo* assembly and subsequent BLASTN analysis using the in-house developed database was the superior approach to obtain results faster compared to the other two. Additionally, the 16S–23S rRNA encoding region NGS-based method was superior in distinguishing bacterial species and in the identification of additional species per sample, not detected by conventional methods.

The initial evaluation study of the sequencing protocols demonstrated the potential use of a shorter read length sequencing kit compared to the longer ones. Even though the number of sequencing reads generated was lower with the 300-cycles kit than the 600-cycles kit, it provided a similar resolution at the bacterial species identification level as the other two kits, with the advantage of being much faster. The use of a faster sequencing workflow may improve the implementation of the appropriate antimicrobial therapy by providing a faster diagnostic answer. Therefore, this approach was chosen for the sequencing of the following samples.

The data analysis of the mock community sample (Supplementary Table S5) showed that the mapping approach was much less sensitive and specific than the other two data analysis approaches. The lower specificity might be explained by the nature of the mapping approach, which allows for a lower degree of homology (80% similarity in at least 50% coverage). This could be improved by changing to more stringent analysis parameters, however, this would have affected the sensitivity of a method that already underperformed, as two species could not be identified. The OTU clustering and *de novo* assembly followed by BLAST approaches performed the species identification with the same accuracy.

During the analysis, the main challenge was the presence of contaminating species. All species detected in the negative controls (Supplementary Tables S2–S4) have been previously described as contaminants of sequencing-based analysis stemming from DNA extraction kits and other laboratory reagents (Salter et al., 2014). These species were highly abundant in samples with low abundant infectious microorganisms and in negative samples, whereas they were identified in relative lower abundancy in true positive samples (conventional positive samples). This suggests that highly abundant contaminants might be masking low abundant infectious microorganisms in some samples. *C. acnes* was found in the majority of samples in low abundancy, especially when using the *de novo* assembly and BLAST approach (samples 1, 2, 10, 11, 15, 17). In addition to being a common bacterium of the human skin and a contaminant from laboratory reagents or the environment (Salter et al., 2014; Mollerup et al., 2016), *C. acnes* has been also described as a cause of infective endocarditis (IE) (Sohail et al., 2009), and prosthetic joint infections (Zeller et al., 2007). Most of our samples (*n* = 25) were from patients with a diagnosis of IE established by an expert panel, taking into consideration all information available, and therefore, we did not immediately filter out the *C. acnes* from the clinical samples, in order not to disregard this pathogen as a cause of infection. Instead, we defined cut-off values to distinguish contaminants introduced during sample handling from an infectious microorganism. Only *C. acnes* found in abundancies above the calculated cut-off level were included, while others below the cut-off level were discarded. Yet, like in our study, we would like to highlight that these results should be interpreted in light of other clinical data available.

Another challenge of 16S–23S rRNA encoding region NGS data analysis was the absence of a database specific for the 16S–23S rRNA encoding region. By creating an in-house developed database, we aimed to overcome the bias of data analysis introduced by using the public 16S rRNA gene databases. On the other hand, the database should be as complete as possible to identify all relevant bacterial pathogens. For this reason, we compared the sequences present in our database with the emerging infectious diseases and pathogens in the Netherlands published by the Dutch National Institute for Public Health and the Environment (RIVM) (de Gier et al., 2017) and emerging diseases and pathogens published by the National Institute of Allergy and Infectious Diseases (NIAID) in the United States (NIAID, 2018). Emerging Infectious Diseases/Pathogens | NIH: National Institute of Allergy and Infectious Diseases) (see Supplementary Table S6). This demonstrated that our database contains pathogens that are common in the Netherlands, while as for pathogens that are common in the United States, some species are missing (e.g., *Rickettsia prowazekii, Anaplasma phagocytophilum, Borrelia miyamotoi, Ehrlichia chaffeensis*, and *Ehrlichia ewingii*). We also looked at the number of occurrences (≤20) per species found in the database and compared it with the NCBI genome database to see how many genome assemblies were available for a specific species. This revealed that many species with few occurrences in our local database also had few genome assemblies available. The NCBI genome database provides completely sequenced genomes and also sequences that are incomplete, and these can be at the contig-, scaffold- or chromosome-level (Kitts et al., 2016). This has the disadvantage of not always being possible to find a 16S–23S rRNA encoding region amplicon due to incomplete sequencing assemblies available. On the other hand, some species had many genome assemblies available which means that more 16S–23S rRNA encoding region sequences can still be added to the database, despite the considerable number of sequences (23,439 entries) already present. As new species are identified, especially from anaerobes, more and more sequences need to be added and updated, as well. Also, the same species might have different number of 16S–23S rRNA encoding regions and different ITS sequences, hence the database should be broad enough to represent different strains of the same species. A comparison of the in-house developed local database to the NCBI database revealed that the BLASTN analysis on the local database was at least as accurate as the BLASTN analysis on the NCBI database in identifying bacterial species despite the differences in identification for sample 18 and 26, which demonstrated a technical challenge and an interpretation challenge for *de novo* assembly and BLAST. With *de novo* assembly, the contigs generated are sometimes too short (<1 kb) and only include part of the 16S or 23S rRNA genes. In sample 18, the contig with 454 bp was identified as *Herbaspirillum* sp. using the NCBI database and was matching the partial sequence of the 16S rRNA gene with a similarity score of 96.47%. In the same analysis, second and third hit matching the same contig was *Massilia* sp. and *Bordetella* sp. with a similarity score of 96.25% and 96.03%, respectively. As *Herbaspirillum* sp. is a potential contaminant (Salter et al., 2014), and its association to human infection has not been described so far, we discarded it in our analysis. On the other hand, the same contig was identified as *Bordetella* sp. (96% similarity score) using the local database and this species could be a potential pathogen. The limitation of our local database is that we do not have as much sequences as NCBI has, since our local database contains only entire sequences of the 16S–23S rRNA encoding region whereas the NCBI database contains also partial sequences of the 16S rRNA or 23 rRNA regions. This explains the difference between the two methods, since the closest reference in our database was the *Bordetella* sp. which was only identified as third hit in NCBI. However, one cannot discard the potential presence of this species in the sample, since the similarity scores were very similar (all around 96%). Furthermore, mapping approach also identified *Bordetella* species but with a very low abundance, below the cut-off value and OTU clustering did not identify *Bordetella* species at all. To confirm the presence of the pathogen, one should use another methodology, e.g., *Bordetella* specific PCR. In sample 26, an additional species *Undibacterium oligocarboniphilum*, which has been described as a common contaminant of DNA extraction kits and other laboratory reagents (Salter et al., 2014), could not be identified as the local database was lacking the corresponding 16S–23S rRNA encoding region sequences for this species. Thus, this gap has no negative clinical consequences.

Conventional microbial diagnostic methods of culturing and 16S rRNA gene Sanger sequencing were used as reference to evaluate the results of all three approaches and differences between them. In this analysis, the 16S rRNA gene Sanger sequencing identified sample 26 as *Streptococcus mitis* although the 16S–23S rRNA encoding region NGS identified this sample as *Streptococcus sanguinis* in all three data analysis approaches. *S. sanguinis* belongs to the “mitis group” of the *Streptococcus* genus (Jensen et al., 2016). Based on the 16S rRNA gene, species of the *S. mitis* group display considerable sequence similarity making it difficult to distinguish them from each other (Lal et al., 2011; Jensen et al., 2016). Since the 16S–23S rRNA encoding region provides higher sequence variability, this approach exhibited higher resolution in distinguishing species having high sequence similarities on their 16S rRNA gene. Furthermore, mapping and OTU clustering not only identified the *S. sanguinis* as the most abundant species yet also other *Streptococcus* spp. (Table 2). This suggests that the OTU clustering and mapping approaches were less discriminative at the species level compared to the *de novo* assembly and BLAST approach. Whilst chimeric assemblies can be created when more than one species of a certain genus is present in one sample (as in sample 26). However, there is still no objective way to overcome this problem. One way would be to do supervised assemblies, however that would require previous information about the taxonomic content of the sample and it would still not completely overcome the problem if two species are highly similar. On the other hand, the OTU clustering approach overcome the limitation introduced by the lack of well-characterized reference sequences at the species level. Given that OTU clustering does not require prior information of a reference taxonomy to cluster query sequences into OTUs, it is particularly advantageous to analyze less well characterized microbes (Chen et al., 2013).

The mapping approach identified the low abundant *Gemella* sp. in sample 3 while OTU clustering, *de novo* assembly and conventional methods did not. *Gemella* sp. are facultative human bacterial pathogens, causing fatal infections related to IE both in pediatric and adult individuals (Purcell et al., 2001; Yang and Tsai, 2014; Jayananda et al., 2017). As sample 3 is a tissue sample taken from a patient suspected of having IE, it seems reasonable to find the *Gemella* sp. in this sample. However, it should be considered that the default similarity and length fraction parameters defined to assign a read to a specific species were 0.8 and 0.5, respectively, meaning that at least 50% of the alignment had at least 80% sequence similarity. These default parameters were much more flexible than the ones defined for BLASTN and OTU clustering, in which the reads were assigned to a specific species only with a similarity score of 99%. This might be the reason why the *Gemella* sp. identified by mapping could not be detected by the OTU clustering nor the *de novo* assembly. However, changing the similarity score for the last two approaches would have resulted in lower specificity, which is not desirable either.

Besides *Gemella* sp., there are other bacterial species known to cause IE, namely *Staphylococcus* spp., *Streptococcus* spp., and *Enterococcus* spp., which are considered as the top three most frequent etiologic agents in both native and prosthetic valve IE (Munita et al., 2012). As described before, in sample 10, the 16S–23S rRNA encoding region NGS approach could identify a low abundant *Enterococcus faecium* (Table 2). Also *T. whipplei*, which is the causing agent of an often predominantly gastrointestinal illness, Whipple’s disease, has been shown to cause IE (Goldenberger et al., 1997; Gubler et al., 1999; Geissdörfer et al., 2012). In an observational study, *T. whipplei* was reported as the fourth most common pathogen causing 6.3% of culture-negative endocarditis cases, determined by the 16S rRNA gene amplification and subsequent sequencing (Geissdörfer et al., 2012). In the present study, 16S rRNA gene Sanger sequencing identified *T. whipplei* in samples 10 and 17, that are tissue samples taken from patients with IE, while the 16S–23S rRNA encoding region NGS could not. Even though there were 12 sequences of the 16S–23S rRNA encoding region of this species present in the local database. When we aligned the PCR primers to those sequences, we observed that the primers did not target the 16S–23S rRNA encoding region of *T. whipplei*, meaning that amplification and subsequent sequencing of the 16S–23S rRNA encoding region of this species did not occur. This is a pitfall of the current method to diagnose *T. whipplei*-associated diseases from patients’ samples (CSF, blood, joint fluid/synovia, potentially gut mucosa) and could only be overcome by designing new and/or adding primers. Another solution would be to sequence the whole microbial DNA directly from patient samples by shotgun metagenomics, overcoming the primer-associated challenges of a targeted NGS approach. Nonetheless, analysis of the large metagenomics data, more complicated than the targeted NGS, requires further technological and bioinformatics developments to be implemented in diagnostic laboratories (Deurenberg et al., 2017).

Although the number of samples tested in this study was too low to statistically evaluate the significance of these approaches compared to conventional test results, it did provide similar results in most cases (80% concordance with conventional methods) or even superior in other cases. The identification of additional species per sample, not detected by conventional methods, demonstrates the potential of the 16S–23S rRNA encoding region NGS-based method in characterizing multiple bacterial species, particularly in polymicrobial samples. Additionally, the 16S–23S rRNA encoding region NGS-based method was superior in distinguishing bacterial species. This is most likely due to the fact that the 16S–23S rRNA encoding region has a higher resolution and more sequence variability compared to the 16S rRNA gene. Faster and less laborious bioinformatics analysis provided by *de novo* assembly and BLAST approach using an in-house database argues for the implementation of the 16S–23S rRNA encoding region NGS-based method for improved diagnostics by means of reducing the time until administration of the appropriate antimicrobials. Additionally, fast growing long-read sequencing platforms, have the potential in the future to reduce even further the time for diagnosis, by providing the possibility for real-time sequencing and probably reducing the need for assembly.

## Conclusion

The higher resolution at the species level identification provided by 16S–23S rRNA encoding region NGS makes its use in routine diagnostic microbiology potentially attractive. Particularly, data analysis is one of the most important steps of a diagnostic workflow, which requires an optimal pipeline for the interpretation of the sequencing data in a short time. This study demonstrates that *de novo* assembly and subsequent BLASTN analysis using an in-house developed database compared to OTU clustering and mapping approaches is the most accurate and fastest approach for identification of bacterial pathogens. Yet, OTU clustering should be considered as a second approach if no pathogen species are identified. Although the in-house developed publicly available database has been shown to be robust enough to identify and distinguish relevant bacterial species, it should be continuously updated to represent more currently relevant or emerging pathogens. In conclusion, advancements of the 16S–23S rRNA encoding region NGS-based method along with the subsequent data analysis of *de novo* assembly and BLAST using a 16S–23S rRNA encoding region database has the potential to be integrated into the routine diagnostic workflow by providing a more accurate and rapid microbial diagnosis.

## Author Contributions

NC, JR, BS, MK-S, EZ, and GW conceived and designed the experiments. NP, SG-C, and BD performed the experiments. NP, SG-C, and HW analyzed the data. AF and JR contributed with reagents, materials, and analysis tools. NP and SG-C wrote the draft manuscript. All authors revised the manuscript.

## Conflict of Interest Statement

The authors declare that the research was conducted in the absence of any commercial or financial relationships that could be construed as a potential conflict of interest.

## Footnotes

1 https://www.ncbi.nlm.nih.gov/genome/

2 http://rast.nmpdr.org

3 ftp://ftp.ncbi.nih.gov/pub/taxonomy/

4 https://github.com/zyxue/ncbitax2lin/

5 https://figshare.com/s/729b346eda670e9daba4

6 https://blast.ncbi.nlm.nih.gov/Blast.cgi

## Supplementary Material

TABLE S1 Parameters.

TABLE S2 BLAST.

TABLE S3 Mapping.

TABLE S4 OTU Clustering.

TABLE S5 Mock Community.

TABLE S6 Emerging Infectious Diseases.
